# The effect of central lymph node dissection on the prognosis of cN0 papillary thyroid microcarcinoma: a mid-term follow-up study

**DOI:** 10.1186/s12902-023-01375-6

**Published:** 2023-05-29

**Authors:** Xiaozhang Xie, Jianwei Deng, Bingxing Zheng, Linkun Zhong, Jianhang Miao

**Affiliations:** The Second Department of General Surgery, Zhongshan City People’s Hospital, No. 2, Sunwen East Road, Zhongshan, Guangdong528403 China

**Keywords:** Papillary thyroid microcarcinoma, Central lymph node dissection, Recurrence

## Abstract

**Background:**

To investigate the effect of central lymph node dissection on the prognosis of patients with papillary thyroid microcarcinoma (PTMC) without clinical lymph node metastasis (cN0).

**Methods:**

According to the inclusion and exclusion criteria, 462 patients with cN0 PTMC underwent surgery in the Second Department of General Surgery, Zhongshan City People’s Hospital from January 1, 2007, to June 31, 2017. They were divided into two groups: the undissection group (262 cases) and the dissection group (170 cases). A comparison was made between the two groups in terms of postoperative complications, recurrences, metastases, etc., as well aslymph node metastasis risk factors in the central region of cN0 PTMC.

**Results:**

There was no lymphatic leakage or death in all patients after the operation. In the dissection group, 64 cases (37.6%) of central lymph node metastasis were found after the postoperative pathological examination. The undissection group was followed up for (92 ± 28.7) months, and the dissection group was followed up for (86 ± 25.4) months (t=-2.165, P = 0.031). In the two groups, there were no lung metastases, bone metastases, or other distant metastases during the follow-up period. In the undissection group, there were 7 cases, while in the dissection group, there were just 2. Recurrence rates between the two groups did not differ significantly (χ^2^ = 0.126, P = 0.169); Similarly, disease-free survival curves did not differ significantly (χ^2^ = 2.565, P = 0.708). Hypoparathyroidism and Hypocalcemia also had no difference between the group. In comparison to the undissection group, the capsular invasion rate (P = 0.026), calcification rate(P < 0.001) incidence of postoperative hoarseness (P = 0.017), and hand and foot numbness rate (P < 0.001) were all considerably greater in the dissection group. Multivariate research revealed that capsular invasion (OR = 9.42, P = 0.002), multifocal (OR = 24.57, P < 0.001), and tumor diameter > 5 mm (OR = 5.46, P = 0.019) were the independent risk factors for central lymph node metastasis in cN0 PTMC.

**Conclusions:**

Thyroidectomy alone is safe for cN0 PTMC, but longer-term follow-up is still required for changes in central lymph nodes. For cN0 PTMC patients with tumor diameter > 5 mm, multifocal, and capsular invasion, central lymph node metastasis is more likely to occur. Comprehensive evaluation and individualized and precise treatment are essential.

## Introduction

Since the advent and widespread use of ultrasound and fine-needle aspiration biopsy in recent years, thyroid cancer incidence rates have been rising quickly worldwide year after year. The incidence of thyroid cancer in the United States has tripled over the past 25 years, largely as the discovery of papillary thyroid microcarcinomas (tumors < 1 cm in diameter)[1].

The vast majority of PTMCs have no obvious clinical symptoms, some PTMCs progress slowly and show “indolent” characteristics, but some PTMCs are more invasive [2], and there are currently no definite clinical or laboratory indicators to distinguish “indolent” PTMCs from invasive PTMC [3]. Some scholars [4–7] believe that one of the risk factors for invasive PTMC is central lymph node metastasis. Although PTMC is early-stage cancer, most PTMCs have a good prognosis. Therefore, there is still considerable controversy in the medical community on whether central lymph node dissection should be performed in PTMC [8]. This study recruited 462 PTMC patients admitted to the Second Department of General Surgery of Zhongshan City People’s Hospital for a retrospective study to explore the effect of central lymph node dissection on the prognosis of cN0 PTMC patients.

## Methods

### Study design and population

A total of 462 patients with cN0 PTMC who underwent thyroid cancer surgery in our hospital from January 1, 2007, to June 31, 2017, were retrospectively collected. There were 370 females and 92 males, ranging in age from 12 to 75 (42.13) years; 299 instances exhibited calcification, while 163 did not; 307 cases had a single lesion, while 155 cases had multifocal lesions. All patients had undergone total thyroidectomy and radioactive iodine ablation. All operations were performed by the chief physician with more than 15 years of surgical experience in our hospital, and the operation followed refined capsule anatomy techniques. According to whether the central lymph node dissection was performed or not, they were divided into the undissection group (292 cases) and the dissection group (170 cases).

Age at diagnosis, sex, maximum tumor diameter (MTD), multifocality, capsular invasion, calcification, surgical complications, hypocalcemia, and hypoparathyroidism was among the baseline and clinical variables we retrieved.

The inclusion and exclusion criteria are as followed.

**Criteria for inclusion of cases**.

1. Pathologically confirmed papillary thyroid carcinoma.
2. Tumor diameter ≤ 10 mm.
3. No palpable cervical lymphadenopathy on preoperative physical examination.
4. cN0: Preoperative color Doppler ultrasound and contrast-enhanced neck CT showed no evidence of central or lateral neck lymph node metastasis.
5. Cases receiving regular follow-up. The minimum duration of follow-up will be one year from baseline.

**Criteria for exclusion of cases**.

1. Papillary thyroid cancer with cervical lymph node metastases was found in the preoperative examination.
2. Cases with no regular follow-up of less than one year.

### Follow-up and observation indicators

Regular follow-up was conducted by subsequent consultation with doctors. The nodal metastasis status and the tumor recurrence were recorded. Regular review of thyroid function was recorded including thyroglobulin and thyroglobulin antibodies. Recurrence was defined as relapse of the tumor after completion of the primary treatment. All patients underwent examination of thyroid color Doppler ultrasound, contrast-enhanced CT, whole body bone imaging nuclear scan, etc. at follow-up.

### Potential confounders

A priori choices of potential confounders were made. As for the potential confounders, they included eating habits, smoking, and the total number of years with malignancies were all demographic confounders. Hypertension, stroke history, diabetes, anemia, body mass index, and prescription nonsteroidal anti-tumor medications were all medical confounders. Medical history, medications, and health-related behaviors were gathered through a clinical interview.

### Statistical methods

Software versions SPSS 23.0 and Excel 2016 were used for statistical analysis and data collecting, respectively. Measurement data that followed the normal distribution were expressed as mean ± standard deviation (x¹ ± s) and analyzed using an unpaired t-student test; enumeration data were expressed as a percentage and used χ2 test. Univariate analysis was performed using the χ2 test, and multivariate analysis was performed using the logistic regression model. Survival curves were made using GraphPad Prism 7 software, and Kaplan-Meier analysis and log-rank test were used for survival analysis. P < 0.05 were considered significant.

## Results

### Participants

There was no lymphatic leakage or death in all patients after the operation. Postoperative pathological examination in the dissection group showed 64 central lymph node metastasis(pN1) cases (37.6%), and 106 cases (62.4%) without central lymph node metastasis (pN0). The undissection group was followed up for (92 ± 28.7) months, and the dissection group was followed up for (86 ± 25.4) months. There was a significant difference in follow-up time between the two groups (t = 2.165, P = 0.031).

### Postoperative and follow-up results

During the follow-up period, there were no bone metastases, lung metastases, or other distant metastases in either group. In the undissection group, there were 7 cases of recurrence, and the recurrence time ranged from 1 to 6 years after surgery. Four of them had ipsilateral lateral lymph node metastases and underwent lateral lymph node dissection; one had ipsilateral central lymph node metastases and underwent central lymph node dissection; and one had ipsilateral region III lymph node enlargement and color Doppler ultrasound evidence of recurrence, leading to the decision to carry out follow-up observation; the other case was isthmus papillary carcinoma with recurrence of right papillary carcinoma 2 years after the operation, and right lobectomy and central area were performed Lymph node dissection. Two cases of recurrence occurred in the group that received lateral neck lymph node dissection; both of these cases were metastatic to the ipsilateral cervical lymph node. Between the two groups, there was no discernible difference in the recurrence rate (χ^2^ = 0.126, P = 0.169, Table 1). In neither group did the patient die. Both groups of patients experienced transitory symptoms like momentary hoarseness, coughing after drinking water, and numbness in the hands and feet (Table 1), Hypoparathyroidism and Hypocalcemia also had no difference between the group, and no permanent complications occurred.

**Table 1:** Comparison of general data and clinicopathological factors between the non-dissection group and the dissection group

Clinical features	Undissection(n, %)	dissection(n, %)	χ2 or t	P value
**Gender**				
Male	54(18.5)	38(22.3)	1.004	0.189
Female	238(81.5)	132(77.7)		
**Age**				
< 55	233(79.8)	148(87.1)	3.291	0.057
≥ 55	59(20.2)	22(12.9)		
**Multifocal**	89(30.4)	66(38.8)	2.439	0.118
**Bilateral**				
Positive	27(9.2)	29(17.1)	6.156	0.011
Negative	265(90.8)	141(82.9)		
**Capsular invasion**				
Positive	45(15.4)	44(25.9)	4.98	0.026
Negative	247(84.6)	126(74.1)		
**Tumor diameter**				
≤ 5 mm	148(50.7)	53(31.2)	16.637	< 0.001
> 5 mm	144(49.3)	117(68.8)		
**Calcification**				
Positive	165(56.5)	134(78.8)	17.362	< 0.001
Negative	127(43.5)	36(21.2)		
BRAF*				
Positive	122(62.9)	70(41.7)	0.014	0.491
Negative	72(37.1)	98(58.3)		
**Postoperative complications**				
Hoarseness	4(1.4)	9(5.3)	6.051	0.017
Humbness	23(7.9)	46(27.1)	31.119	< 0.001
Choking	1(0.3)	4(2.4)	4.048	0.064
Lymphatic leakage	0	0	0	0
Recurrence	7(2.4)	2(1.1)	0.126	0.169
**Hypoparathyroidism**				
Yes	50(17.1)	33(19.4)	0.382	0.537
No	242(82.9)	137(80.6)		
**Hypocalcemia**				
Yes	55(18.8)	35(20.6)	0.398	0.528
NO	247(84.6)	135(79.4)		

*BRAF gene test was carried out in our hospital after June 2015, only some cases were counted

### Comparison of clinicopathological factors between the undissection group and dissection group

There was no significant difference in gender, age, multifocal, BRAF, the incidence of postoperative drinking water choking, and recurrence rate between the undissection group and the dissection group ( P > 0.05), while the capsular invasion rate (P = 0.026), calcification rate (P < 0.001), the incidence of hoarseness (P = 0.017), and incidence of numbness of hands and feet (P < 0.001) in the dissection group were significantly higher than those in the undissection group (see Table 1).

Gender, age, multifocal, BRAF, the incidence of postoperative drinking water choking, and recurrence rate were not significantly different between the undissection group and the dissection group (P > 0.05), but capsular invasion rate (P = 0.026), calcification rate (P0.001), the incidence of hoarseness (P = 0.017), and incidence of numbness in the hands and feet (P0.001) were significantly higher in the dissection group than in the undissection group (see Table 1).

### Influencing factors of central lymph node metastasis in cN0 PTMC and survival analysis

**Fig. 1:**
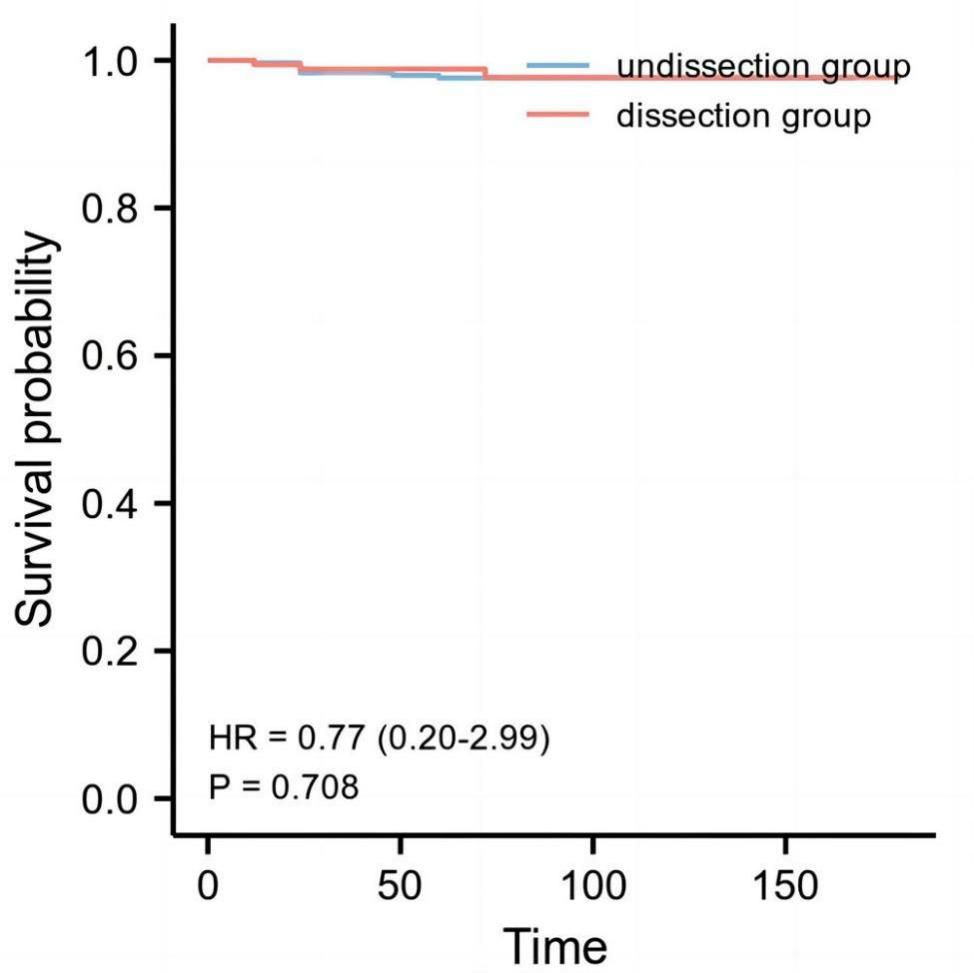
The disease-free survival curve of the dissection group and the undissection group

Central lymph node metastasis was linked to bilateral (P = 0.037), multifocal (P = 0.023), tumor diameter (P = 0.032), and capsular invasion (P0.001) in cN0 PTMC (Table 2). For lymph node metastasis in the middle of the neck, multifocal (OR = 24.57, P0.001), tumor diameter > 5 mm (OR = 5.46, P0.001), and capsular invasion (OR = 9.42, P0.001) were independent risk factors (Table 3). The undissection group and the dissection group did not have a statistically different disease-free survival curve (χ^2^ = 2.565, P = 0.109, Fig. 1).

**Table 2:** Univariate analysis results of central lymph node metastasis in cN0 PTMC (n, %)

Clinical features	pN0(n = 106)	pN1(n = 64)	χ2	P
**Gender**				
Male	23(21.7)	15(23.4)	0.07	0.850
Female	83(78.3)	49(76.6)		
**Age**				
< 55	90(84.9)	58(90.6)	1.159	0.350
≥ 55	16(15.1)	6(9.4)		
**Multifocal**				
Positive	20(18.9)	35(54.7)	8.54	0.023
Negative	86(81.1)	29(45.3)		
**Bilateral**				
Positive	13(12.3)	16(25)	4.575	0.037
Negative	93(87.7)	48(75)		
**Tumor diameter**				
≤ 5 mm	37(34.9)	17(26.6)	1.825	0.032
> 5 mm	69(65.1)	47(73.4)		
**Capsular invasion**				
Positive	11(10.4)	36(56.3)	39.064	<0.001
Negative	95(89.6)	28(43.7)		
**BRAF**				
Positive	43(40.6)	29(45.3)	0.368	0.631
Negative	63(59.4)	35(54.7)		
**Calcification**				
Positive	83(78.3)	54(84.4)	0.941	0.424
Negative	23(21.7)	10(15.6)		
**hypocalcemia**				

*BRAF gene test was carried out in our hospital after June 2015, only some cases were counted

**Table 3:** multivariate analysis results of central lymph node metastasis in cN0 PTMC

Variate	*β*	standard error	*χ* ^2^	*OR*	P
**Gender**	0.243	0.467	0.27	0.27	0.603
**Age**	0.375	0.408	0.847	0.85	0.357
**Multifocal**	–1.979	0.399	24.565	24.57	< 0.001
**Bilateral**	–0.103	0.429	0.058	0.06	0.81
**Tumor diameter**	–0.969	0.415	5.456	5.46	0.019
**Capsular invasion**	–1.289	0.42	9.416	9.42	0.002
**BRAF**	0.301	0.814	1.351	0.334	0.367
**Calcification**	0.321	0.563	1.379	0.428	0.453

BRAF: v-raf murine sarcoma viral oncogene homolog B1

## Discussions

Differentiated thyroid cancer [9], particularly papillary thyroid microcarcinoma, has been more frequently discovered in recent years thanks to advancements in thyroid ultrasonography technology. It has been reported [10, 11] that the postoperative central lymph node metastasis rate of cN0 PTMC is 30–65%. Li et al. [12] showed that males, age < 45 years old, with tumor diameter > 5 mm, capsular invasion, extracapsular invasion, and single foci located in the lower pole of the gland lobe should be alert to the possibility of central lymph node metastasis; Wang et al. [13] showed that PTMC patients with characteristics such as age < 45 years old and capsular invasion are more likely to develop central lymph node metastasis, therefore prophylactic central lymph node dissection is recommended. Our data showed that multifocality, tumor diameter > 5 mm, and capsular invasion are independent risk factors for central lymph node metastasis. As cN0 PTMC has a high rate of central lymph node metastasis and many factors could affect the occurrence of central lymph node metastasis, whether all PTMCs require central lymph node dissection?

At present, there is still considerable controversy as to whether central lymph node dissection should be performed in cN0 PTMC. The 2015 American Thyroid Association (ATA) guidelines point out that PTMC patients without high-risk factors may be unsuitable for preventive central lymph node dissection after thyroidectomy [14]; however, Japanese guidelines [15] and Chinese expert consensus [16] are more inclined to perform prophylactic central lymph node dissection. The source of the controversy is the indistinguishability of “indolent” PTMC from invasive PTMC. Proponents believe that the rate of lymph node metastasis in the central region of PTMC is relatively high and reach 20.7–62%. When metastasis occurs, the difficulty of reoperation are greatly increased following the significant risks for the complications of surgery. It was reported that the risk of reoperation caused permanent vocal cord paralysis was 4.4%, and the incidence of permanent hypocalcemia reaches 12% [17]. Therefore, preventive central lymph node dissection is required; On the contrary, opponents believe that despite the high central lymph node metastatic rate for cN0 PTMC, tiny lymph node metastases are more common and are therefore not picked up by the current inspection techniques, such as thyroid color doppler ultrasound or improved thyroid CT. And yet, central lymph node dissection will increase the risk of complications, and survival with “tumor” does not affect the prognosis of patients. Dissection of the central lymph nodes as a preventative measure is not recommended. Zheng et al. [18] found that the short-term and long-term efficacy of thyroidectomy combined with isthmus resection in the treatment of PTMC is better than total thyroidectomy combined with central lymph node dissection, thyroidectomy combined with isthmus resection can significantly improve the levels of parathyroid hormone and serum calcium in patients, and reduce complications, recurrence rate, and mortality rate. The findings of Zhang et al. [19] suggest that thyroid lobe and isthmectomy surgery effectively reduce the occurrence of complications and improve the quality of life of patients with no clear central lymph node metastasis before PTMC surgery, can Both surgical methods might be used the main method for the treatment of PTMC. It was reported that PTMC patients with central cervical lymph node dissection are more likely to recur after surgery than those without dissection [20]. Based on the good prognosis of PTMC, some researchers have even started clinical studies on the thermal ablation of PTMC [21, 22]. Consensually, the main surgical indication of lymph node dissection for PTMC: (1) foci diameter > 6 mm; (2) multi-foci carcinoma, especially bilateral carcinoma. For patients with unilateral cancer foci, doctors with rich clinical experience are recommended to perform central lymph node dissection; For patients with bilateral cancer foci, if both lower polar parathyroid glands can be found, double-sided lymph node dissection is recommended to be performed, but if both lower pole parathyroid glands are not found, especially for patients with one cancer in the upper pole, bilateral central lymph node dissection is not recommended. In this study, there was no significant difference in tumor metastasis, recurrence, or prognosis between the two groups. And the occurrence of hypocalcemia and hypoparathyroidism in both groups also showed no difference. However, the incidence of hoarseness and numbness of hands and feet in the dissection group was significantly higher than those in the non-dissection group. These two postoperative complications will reduce the quality of life of patients, and increase the risk of doctors’ practice. However, due to the slow progression of most PTMCs [23] and the limited follow-up time in this study, conclusions may be biased based on the current statistical results. A retrospective follow-up study by Noguchi et al. [24] found that the recurrence rate of patients with a tumor diameter of 1 to 5 mm was 3.3%, while the recurrence rate of patients with a tumor diameter of 6 to 10 mm increased to 14%. Among them, the 30-year cumulative recurrence rate for those aged > 55 years was as high as 40%. Therefore, the metastasis and recurrence of PTMC are determined by multiple factors, which requires us to comprehensively evaluate the conditions of different patients and implement “individualized” precise treatment, rather than all patients being treated according to a unified model. Several studies have shown that active surveillance is an effective first-line therapy for low-risk PTMC[25]. Therefore, active surveillance is necessary to monitor tumor progression.

This study has several limitations. Firstly, our clinical and control samples were limited. Patients were not excluded based on other disease processes. Secondly, the follow-up period was not long. Studies with a large size and longitudinal design are necessary to evaluate the role of dissection in PTMC treatment.

In conclusion, it is undeniable that for some PTMCs with high invasiveness, metastases in the central region, or extensive metastases in the lateral neck, standard cervical lymph node dissection should be performed for such patients, but most cN0 PTMCs are “indolent”, and so are the lymph nodes metastasized in the central region. Therefore, simple resection of the affected thyroid lobe is still a feasible treatment method, with a low recurrence rate and fewer complications, but the final results still require longer-term follow-up.

## Data Availability

The data is available upon reasonable request, Jianhang Miao should be contacted.
